# The use of body surface temperatures in assessing thermal status of hutch-reared dairy calves in shaded and unshaded conditions

**DOI:** 10.3389/fvets.2023.1162708

**Published:** 2023-07-03

**Authors:** Mikolt Bakony, Levente Kovács, Luca Fruzsina Kézér, Viktor Jurkovich

**Affiliations:** ^1^Department of Biostatistics, University of Veterinary Medicine, Budapest, Hungary; ^2^Institute of Animal Sciences, Hungarian University of Agriculture and Life Sciences, Kaposvár, Hungary; ^3^Department of Animal Hygiene, Herd Health and Mobile Clinic, University of Veterinary Medicine, Budapest, Hungary

**Keywords:** dairy calves, heat stress, body surface temperature, rectal temperature, infrared thermometry

## Abstract

The study was carried out in a Hungarian large-scale dairy farm during a 5-day period in hot August weather. Altogether 16 preweaning calves were chosen for the study. An agricultural mesh with 80% shielding was stretched over eight calf cages at 2 m from the ground to shield the cages in their entirety, while eight others were left unshaded. Ambient temperature and relative humidity were measured in 10 min intervals inside and outside one of the hutches in the shaded and unshaded groups during the total length of the study. The rectal temperature of the calves was measured by a digital thermometer every 4 h. Surface temperatures were measured on body parts, in the same intervals as rectal temperature with an infrared thermometer. Measuring sites included: the leg (metacarpus), muzzle, eye bulb, scapula, and ear. Statistical analyses were performed to assess the effects of shading on environmental and body temperatures and to also assess the strength of the association between core, skin and ambient temperatures; to estimate the temperature gradient between body shell and core; to compare the changes in heat dissipation capacity of the different body regions (as represented by temperatures of various sites) with increasing ambient temperature controlling for shaded or unshaded conditions; and to predict the risk of hyperthermia (rectal temperature not lower than 39.5°C) with the CART classification method. The average rectal temperatures suggest that the temperature conditions both in shaded and unshaded groups imposed a severe heat load on the calves. The temperature of the body shell, as represented by skin temperatures, shows a much more significant variation, similar to ambient temperature. As expected, areas that are closer to the core of the body (ear and eye) show less difference from rectal temperature and show a narrower range (lower variance), as more distal regions (leg, scapula) have a wider range. Body surface temperatures are more related to ambient temperature in calves than rectal temperature. The predictive value of infrared body surface temperatures for predicting heat stress or rectal temperature is low.

## 1. Introduction

Increased mortality in the summer (1) suggests that monitoring young calves’ general health and thermal status in hot weather is crucial. Elevated rectal temperature is one indicator used to assess calves’ thermal status (2). However, measuring rectal temperatures requires direct contact with the animals, is time-consuming and does not allow continuous measurements. The duration of handling and restraint, type of thermometer, insertion depth and placement can all have an effect on the results. Methods of no-contact thermometry and thermography have been and are currently being developed (3, 4). Other technologies, for example, a ruminal bolus, may lose efficiency over time or be inaccurate in measurements due to the influence of drinking and the rumen environment in dairy cows and small ruminants (5, 6). However, internal boluses are not yet developed and validated for pre-weaned dairy calves. Intravaginal devices provide measurements in strong agreement with rectal temperatures (7), but they often lead to vaginal irritation. Subcutaneous implants are costly, time-consuming and invasive (6).

While contact thermometry is based on conductive heat transfer, infrared thermometry and infrared thermography measures the emitted radiation. Contactless methods are a noninvasive techniques that might detect body temperature without causing any unnecessary disturbance. Infrared thermography, or using infrared cameras is a relatively new method of measuring body temperature that has gained popularity in recent years. This method provides a visual representation of the animal’s thermal state and can be used to identify potential heat stress problems before they become severe (8). However, infrared cameras can be expensive and require specialized training to operate. Attempts have been made to establish automated systems for temperature monitoring using infrared thermography for health control in swine (9) and cattle (10, 11). Infrared thermometers are other alternative, non-invasive methods of measuring body temperature requiring no physical contact with the calf. The thermometer measures the surface temperature (ST) of the calf’s skin, which can provide an estimate of the internal body temperature. The temperature of the rectal cavity is integrated into the body core, whereas ST relates to the body coat, which is in constant heat exchange with the surrounding environment (12). There are several studies using infrared thermometry and ST in heat stress studies in dairy calves (13–16). Infrared thermometers are a quick and easy way to assess the calf’s thermal state, but they may not be as accurate as rectal temperature measurements, since ST changes according to the environmental conditions (15, 16).

Obtaining skin temperatures are feasible without disturbing the animal and could be easily and quickly performed by the stockperson as part of routine daily observations. Therefore, we wished to investigate how informative skin temperature measurements can be in assessing the thermal status of hutch-reared preweaning calves. Another aspect of knowing the surface temperatures is that it reflects the temperature of the body shell. A cooler body shell allows heat transfer from the core, while a warmer body shell reduces it, resulting in increasing core temperature. The heat dissipation capacity is directly proportional to the area of a surface, its thermal conductivity and the temperature gradient between the surface and core temperature. We aimed to assess the changes in the temperature gradient between the core and shell in relation to ambient temperatures. In summary, we aimed to assess the usefulness of body surface temperature measurements in the following aspects (controlling for shaded/unshaded conditions):

a) strength of association between rectal and surface temperatures,
b) assessing the changes in the magnitude of difference between skin temperatures and rectal temperature in relation to ambient temperature,
c) predictive value of skin temperatures in assessing the risk of hyperthermia.

## 2. Materials and methods

### 2.1. Animals and measurements

The study was approved by the Pest County Government Office, Department of Animal Health (Permit Number: PE/EA/1973–6/2016). Measurements took place in a commercial dairy in Martonvásár, Hungary (47°17′24.3”N 18°48′46.1″E). The farm has a population of 1,000 Holstein Friesian dairy cows. The calves were raised according to the Council Directive 2008/119/EC of 18 December 2008 laying down minimum standards for the protection of calves. The calves were housed outdoors in 1.6 × 1.2 m individual fiberglass-reinforced polyester hutches with a 1.6 m^2^ wired exercise pen, from birth till weaning. The hutches had a rear bedding door on the upper side of rear wall, which was open during the study to ensure proper airflow through the hutch. The calves received 4 L milk replacer from bucket twice daily. Calf starter pellets, alfalfa hay and drinking water were offered *ad libitum*. The study was carried out during a 5-day period in hot August weather. Altogether 16 Holstein Friesian male calves (age = 46.7 ± 2.4 d, body weight = 74.3 ± 2.6 kg) were chosen for the study. An agricultural mesh with 80% shielding was stretched over eight calf cages at 2 m from the ground to shield the cages in their entirety, while eight others were left unshaded. Ambient temperature and relative humidity were measured with Voltcraft DL-181THP devices (Conrad Electronic SE, Hirschau, Germany) in 10 min intervals insideand outside one of the hutches in the shaded and unshaded groups during the total length of the study. Inside the hutch the thermometer was placed in the feeding trough and outside it was placed at 1.5 m height on a pole fixed to the wire fence. The rectal temperature of the calves was measured by a digital thermometer (Digi-Vet SC 12; Jørgen Kruuse A/S, Langeskov, Denmark) every 4 h from 8:00 to 20:00. Surface temperatures (ST) were measured on body parts not exposed to the sun in the same intervals as rectal temperature with an infrared thermometer with a 2-point laser marking (Testo 830 T2, Testo SE & Co. KgaA, Lenzkirch, Germany). Body temperature measurements were carried out outside the hutch. Measuring sites included: the leg (metacarpus), muzzle, eye bulb, scapula, and ear. During measurement, the device was about 10–20 cm from the body surface. The areas were unshaved (16) and shading was applied on the area during measurements. To focus on the effect of shading, the days with cloudy weather was not included in the analysis. The temperature data (both ambient and body temperatures) of the three hottest days (daily average temperatures between 27.3°C – 30.5°C) were used in the analysis.

### 2.2. Statistical analysis

All statistical analyses were performed in software R (17). The cut-off for statistical significance was set at *p* < 0.05.

First, we tested whether the mesh, provided a more favorable thermal environment for the calves. We did this by estimating the effect of shading on the average daily temperature and the diurnal fluctuation of the ambient temperature measured in the outdoor areas and hutch by fitting linear mixed models, respectively, with the time of measurement (time of day: morning, noon, afternoon, evening) and shading, as well as their interaction as fixed factors and day of measurement as a random factor.

Afterwards, the aims mentioned in the introduction were achieved as follows:

a) To assess the strength of the association between core, skin, and ambient temperatures, the repeated measures correlation method (18, 19) was used, which accounts for repeated measurements on the same subject.
b) The temperature gradient between the body shell and core was estimated by calculating the difference between the rectal and skin temperatures. We have investigated the effect of ambient temperature (independent variable) on the temperature gradients (dependent variable) with linear mixed models, with the calf as a random factor.
c) Surface temperatures of body regions identified as most informative about the heat dissipation capacity were then used to predict the risk of hyperthermia [rectal temperature not lower than 39.5°C, after Piccione et al. (20)]. For this purpose a decision tree algorithm method was used. It works by splitting the data into two groups based on one of the explanatory variables (skin temperatures) to achieve maximal homogeneity of the outcome variable (risk of hyperthermia present or not) within the two groups. Then a measure of association is determined between the explanatory variable and the outcome variable in each of the two groups. The cutting point is chosen so that the difference in the association is significantly different between the two separate groups. Such splitting and measure of the association are then applied recursively to each of the respective groups of observations based on another of the explanatory variables. This process is repeated a number of times, selecting different variables to split the data.

## 3. Results and discussion

### 3.1. Effect of shading on the thermal environment of calves

Table 1 displays the average temperatures measured in the morning (8:00), at noon (12:00), in the afternoon (16:00) and in the evening (20:00) in the hutch and outdoor area, respectively of the shaded and unshaded groups.

**Table 1:** Temperatures measured in the outdoor area and inside the hutch at different times of day in shaded and unshaded groups (°C).

	Outdoor area				Hutch			
	Unshaded		Shaded		Unshaded		Shaded	
	Mean	SD	Mean	SD	Mean	SD	Mean	SD
8:00	26.5^a^	2.8	25.9^a^	3.2	26.2^a^	3.0	26.1^a^	2.9
12:00	36.2^b1^	3.3	35.3^b2^	3.2	38.8^b1^	4.2	34.7^b2^	3.5
16:00	40.2^b^	4.3	37.5^b^	2.9	37.8^b1^	2.2	35.9^b2^	2.3
20:00	28.6^c^	1.1	29.0^c^	1.3	29.3^a^	1.2	29.2^a^	1.0

Means with different letters in superscript indicate significant differences (*p* < 0.05) within a column. Means with different numbers in superscript indicate significant differences (p < 0.05) between unshaded and shaded groups at a given site (outdoor area or hutch).

Average temperatures differed with the time of day (*p* < 0.001), being higher at noon and in the afternoon, while shading had a significant effect (*p* < 0.05) on the ambient temperature at 12:00 and 16:00 at both outdoor area and hutch environments.

**Table 2:** Average body temperatures in the groups (mean ± SD).

Temperature (°C)	Unshaded group	Shaded group
Rectal	39.4^a^ ± 0.45	39.3^a^ ± 0.39
Ear	35.2^b^ ± 2.49	34.9^b^ ± 2.32
Eye	35.3^b^ ± 1.56	35.7^b^ ± 1.51
Leg	33.2^c^ ± 2.95	33.1^c^ ± 2.76
Muzzle	32.9^c^ ± 1.88	33.4^c^ ± 1.86
Scapula	34.1^d^ ± 3.15	33.8^d^ ± 2.97

Means with different superscripts indicate a significant difference in the same column (*p* < 0.05).

**Figure 1:**
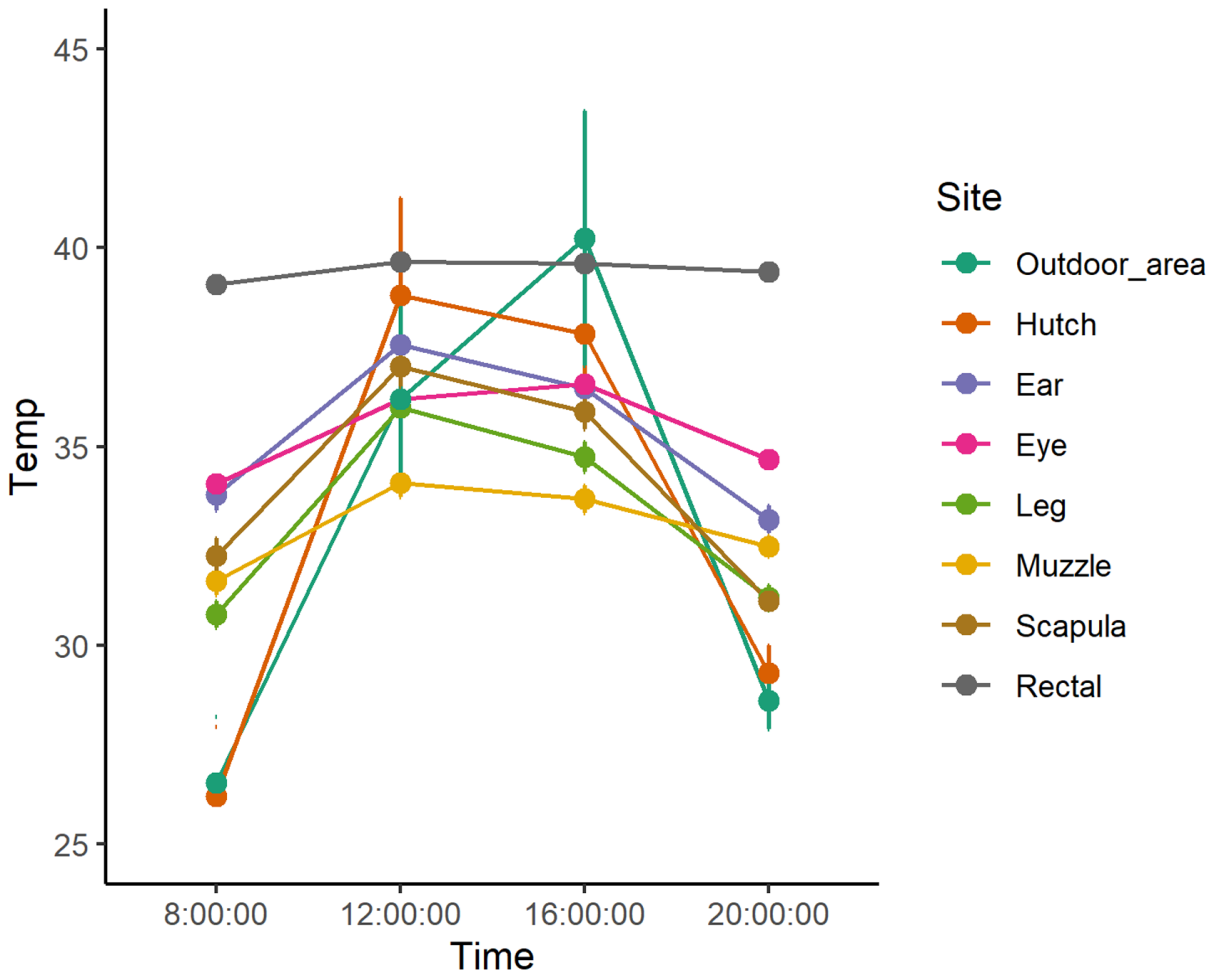
Dry bulb temperature in the outdoor area and body temperatures measured at different body regions during the daytime hours of the three hottest days of the study in the unshaded group.

As expected, the average ambient temperatures in the outdoor area differed significantly by time of day but – except for noon measurements – did not differ, not with the presence or absence of shading. The latter can be explained by the fact that dry bulb thermometers must be shaded. Therefore, they do not measure the heat transferred by solar radiation. However, on one occasion, the thermometers in the unshaded group measured an exceedingly high value above 45°C (Figure 1), which is presumably a consequence of direct sunlight that could have hit the sensor at that time point, which biased the result upwards. Also, skin temperatures did not show a drastic increase that would be expected to follow such a steep increase in ambient temperature (see Figure 1). To account for the outlier nature of this particular value, we have performed comparisons with and without this value. Yet, the significance of differences was similar in both scenarios. Shading reduced the heat accumulation in the outdoor area and the hutch material, therefore providing significantly lower ambient temperatures measured inside the hutch in the hottest period of the days, as it was seen in another study (21). The physiological relevance of the average 2–3°C difference is, however, questionable, as average temperatures in both the hutch were well above the accepted upper critical temperature of dairy calves [26°C; (13, 22)]. Despite the not relevant effects, we have controlled for shading during the rest of the analysis.

### 3.2. Rectal and skin temperatures in the unshaded and shaded groups

**Figure 2:**
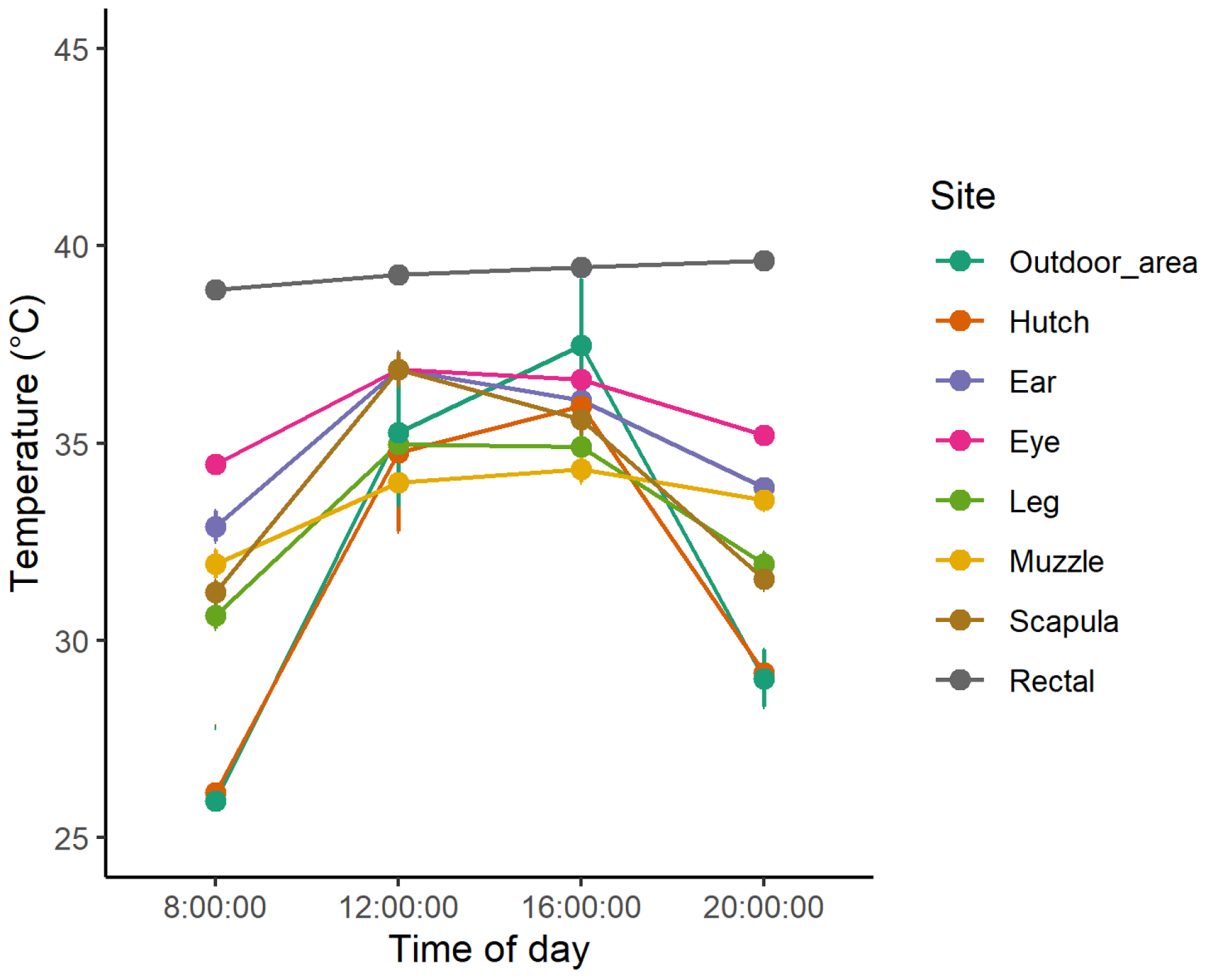
Dry bulb temperature in the outdoor area and body temperatures measured at different body regions during the daytime hours of the three hottest days of the study in the shaded group.

The animal-based temperature measurements and the ambient temperature at the time of measurements in the sunny and shaded groups are displayed in Figures 1, 2 and Table 2.

Though the time points involve only the daytime hours, it still shows the diurnal rhythm of rectal temperature with higher values in the hottest parts of the day and lower values in the morning and evening hours. As compared to the study of Piccione et al. (20), where the core body temperature of preweaning dairy calves showed an average of 38.3°C with an amplitude of 1.4°C, in our study, the temperature values had a similar variation but oscillating around a mean temperature of 39.3–39.4°C, which is 1°C higher than in the study of Piccione et al. (20) that was conducted at lower environmental temperatures (22–28°C). It suggests that the temperature conditions both in shaded and unshaded groups imposed a severe heat load on the calves. We suppose that net shading did mitigate the effect of solar radiation but not to an extent that would cause a significant reduction in rectal temperature., The rectal temperature values were similar to other heat stress studies (15, 16, 23, 24).

The temperature of the body shell, as represented by skin temperatures, shows a much more significant variation, similar to ambient temperature (15) (Table 2). Understandably, as the body surface is the scene of constant heat transfer between the core and the environment, skin temperatures depend on the temperature gradient between the core and the environment (25). Figures 1, 2 display that when the difference between ambient temperature and core body temperature is higher (8:00 and 20:00), skin temperature values measured at different sites tend to scatter more widely; however, when the temperature gradient is smaller (12:00 and 16:00) they tend to show less variability. On most measuring occasions, skin temperatures were above 35°C, limiting the efficiency of heat-flux from the core to the shell and thereby causing a disturbance in maintaining a constant body temperature (25). Figures 1, 2 show that in the hottest hours of the day, most of the body regions had a skin temperature above 35°C, which could explain the average elevated core body temperature.

### 3.3. Association between temperature measures

The correlation of repeated measures between the different temperatures in unshaded and shaded environments is summed up in Table 3. Classification of strength based on correlation coefficients (26).

**Table 3:** Repeated measures correlation between temperature measures in unshaded (no fill) and shaded (gray fill) conditions.

Temperature	Ambient	Rectal	Eye	Ear	Muzzle	Scapula	Leg
Ambient	–	0.61	**0.80**	**0.68**	0.54	**0.76**	**0.77**
Rectal	0.36	–	0.59	0.58	0.55	0.53	0.55
Eye	**0.78**	0.32	–	0.67	0.52	0.69	0.69
Ear	**0.76**	0.31	0.78	–	0.52	**0.82**	**0.81**
Muzzle	0.48	0.43	0.46	0.57	–	0.52	0.66
Scapula	**0.83**	0.18	**0.76**	**0.74**	0.40	–	**0.83**
Leg	**0.84**	0.36	**0.78**	**0.76**	0.57	**0.82**	–

All correlations were significant (*p* < 0.001). Correlations considered to be strong are written in bold.

ST showed a stronger association with ambient temperature than core body temperature. The explanation is that in the measured ambient temperature range, most of the animals could maintain their body temperature close to normothermia. Therefore variability did not exceed that originating from the normal diurnal rhythm (20). Indeed, a high correlation between ST and rectal temperature could only be detected in artificially induced febrile states (27, 28) or inflammation when body temperatures strongly deviate from physiological (11). The correlation between surface and rectal temperatures is influenced by the surrounding environmental conditions, as it is seen in the table. Skin temperatures measured with infrared thermometry are usually significantly lower than rectal temperature (10, 16, 27), and the magnitude of the difference is influenced by the thermal environment (29). According to a recent heat stress study (15) the strongest correlations occurred between ambient temperature on neck, rump and ear of the calves.

### 3.4. Changes in core-shell temperature gradients in relation to ambient temperature

The association between ambient temperature and the gradient between core and surface temperatures are displayed in Table 4.

**Table 4:** Relationship between temperature gradient between core and surface temperatures and ambient temperature in unshaded and shaded groups.

Difference between rectal and body surface temperature	Unshaded	Shaded
*Ear* mean ± SE	4.2 ± 0.2°C	4.4 ± 0.3°C
Range	0.2–8.4°C	0.9–8.2°C
Model fit without outlier	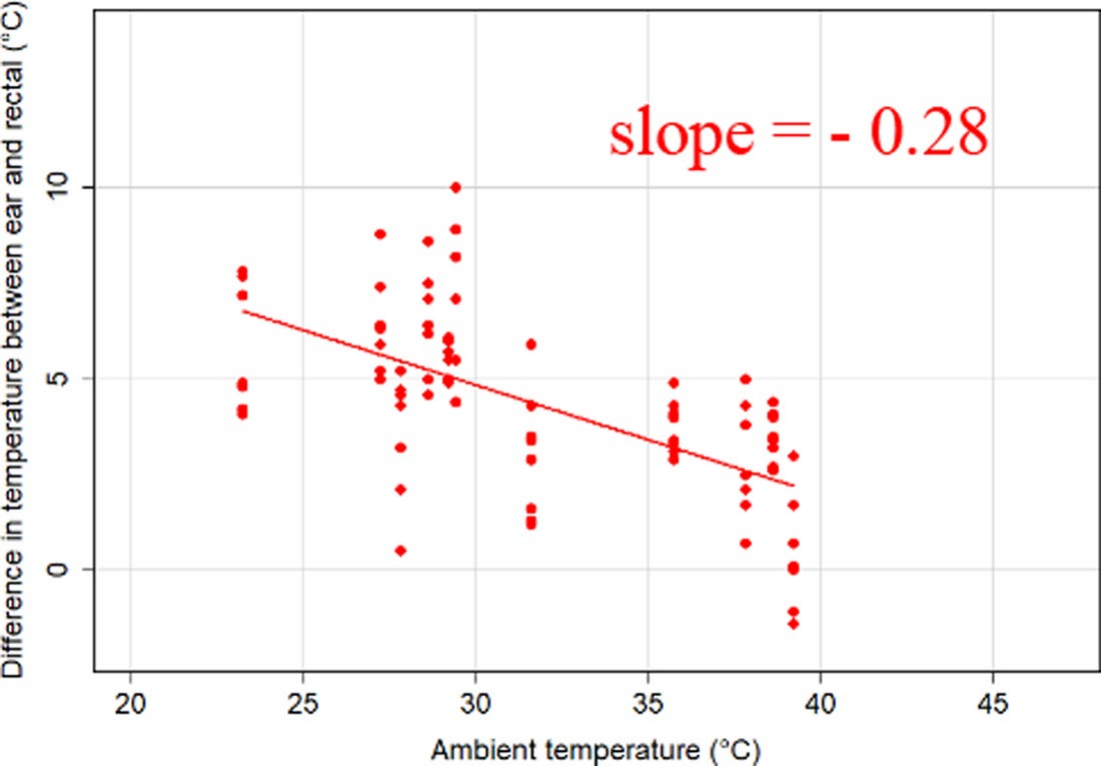	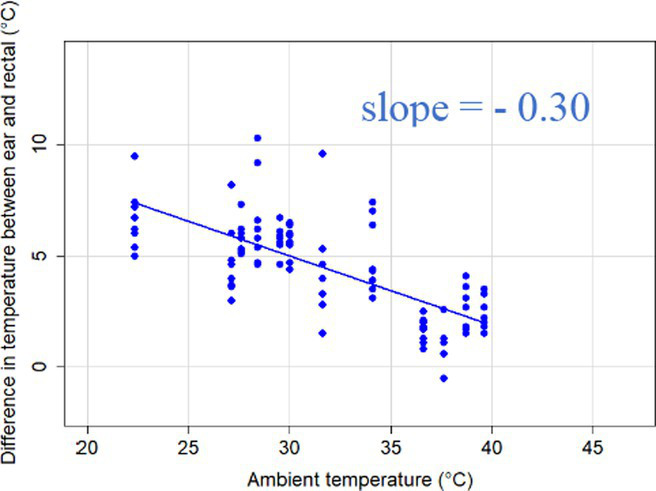
Eye mean ± SE	4.05 ± 0.3°C	3.52 ± 0.3°C
Range	−1.2 – 7.7°C	−0.3 – 6.6
Model fit without outlier	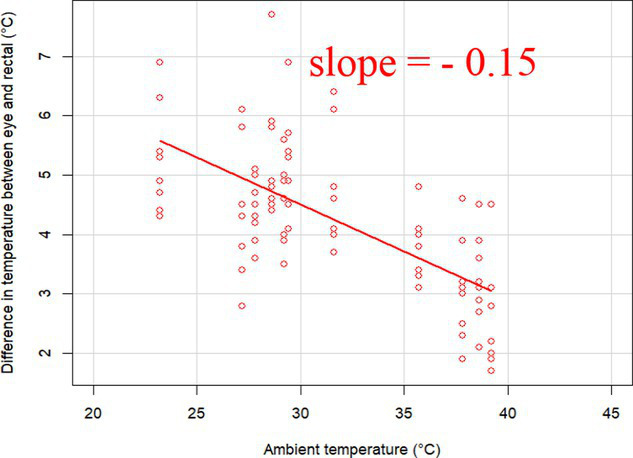	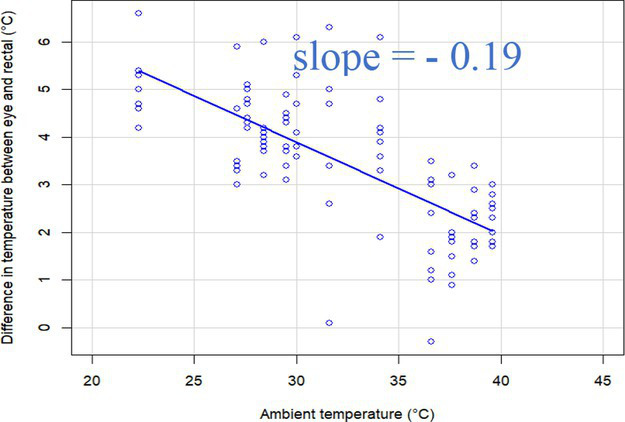
Muzzle mean ± SE	6.5 ± 0.2°C	5.8 ± 0.2°C
Range	3.9–9.0°C	3.2–8.9°C
Model fit without outlier	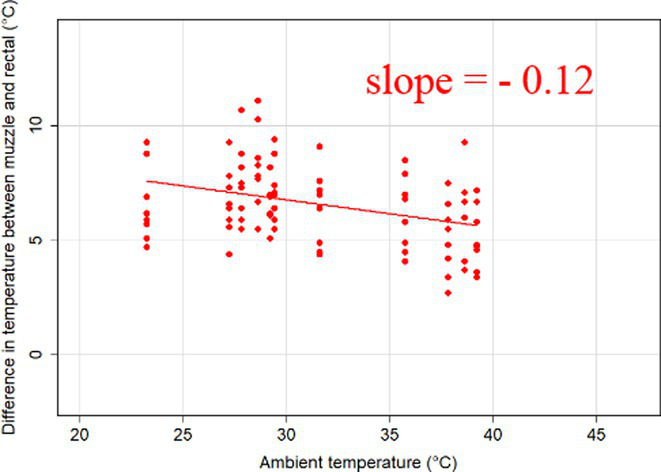	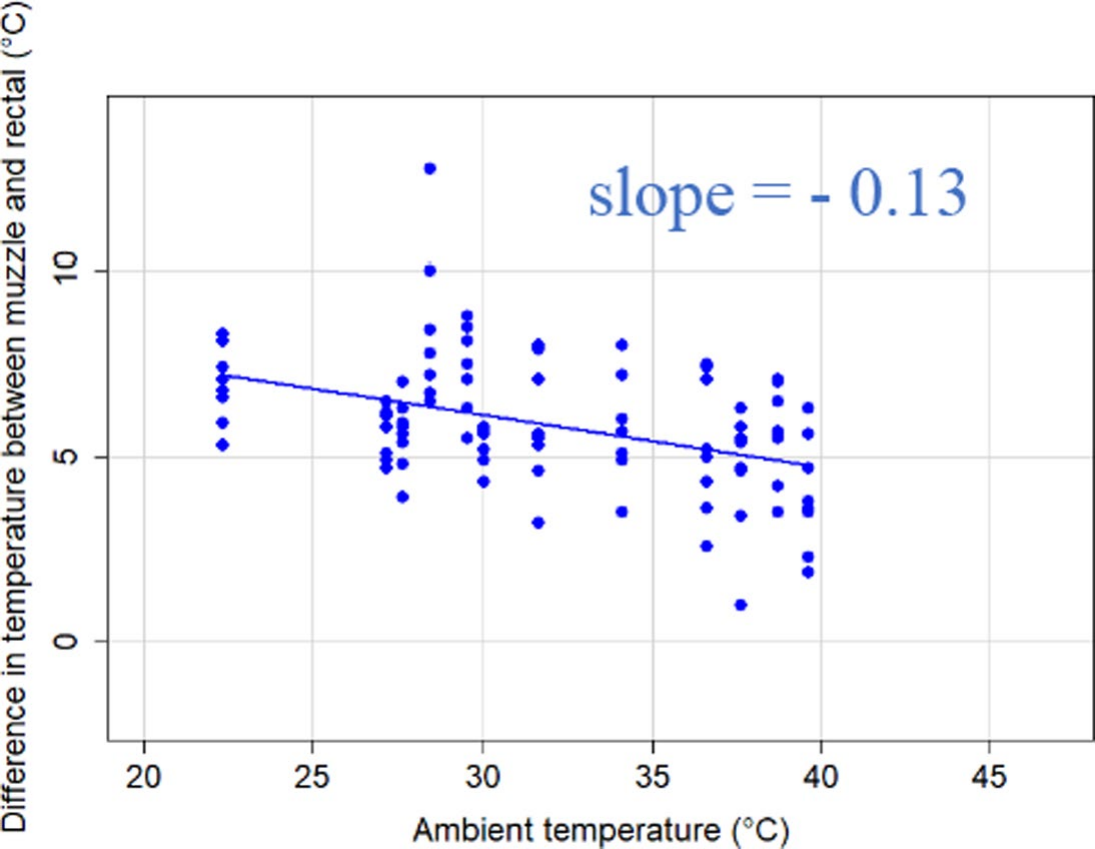
Scapula mean ± SE	5.4 ± 0.3°C	5.5 ± 0.3°C
Range	−0.2–9.2°C	0.6–9.7°C
Model fit without outlier	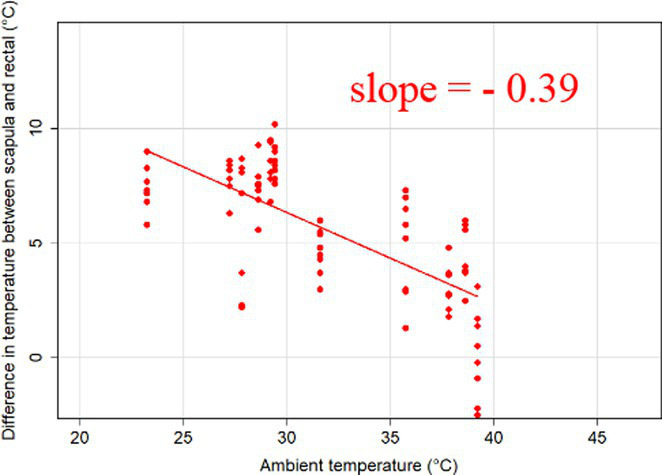	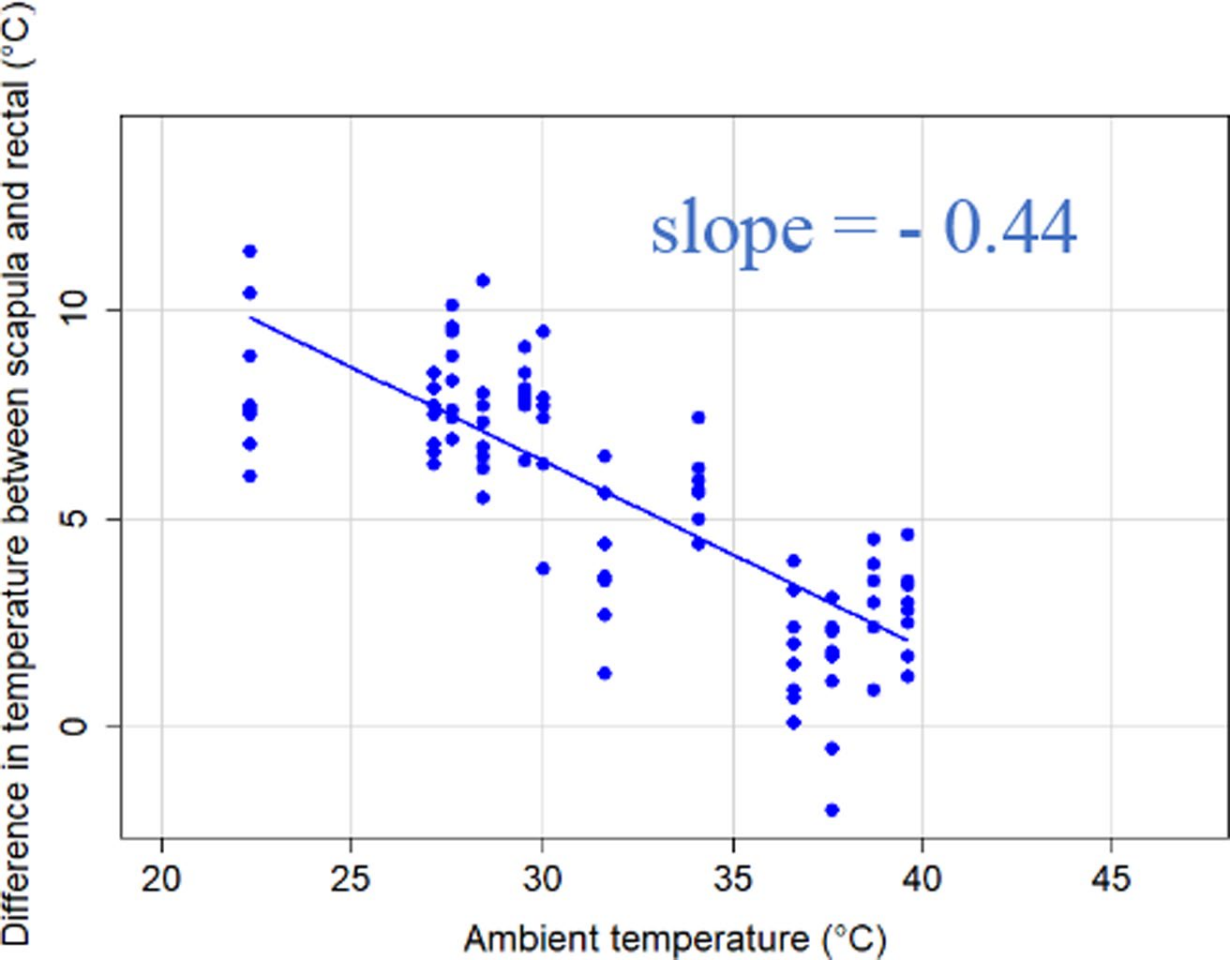
Leg mean ± SE	6.3 ± 0.3°C	6.2 ± 0.3°C
Range	1.4–10.3°C	1.8–9.8°C
Model fit without outlier	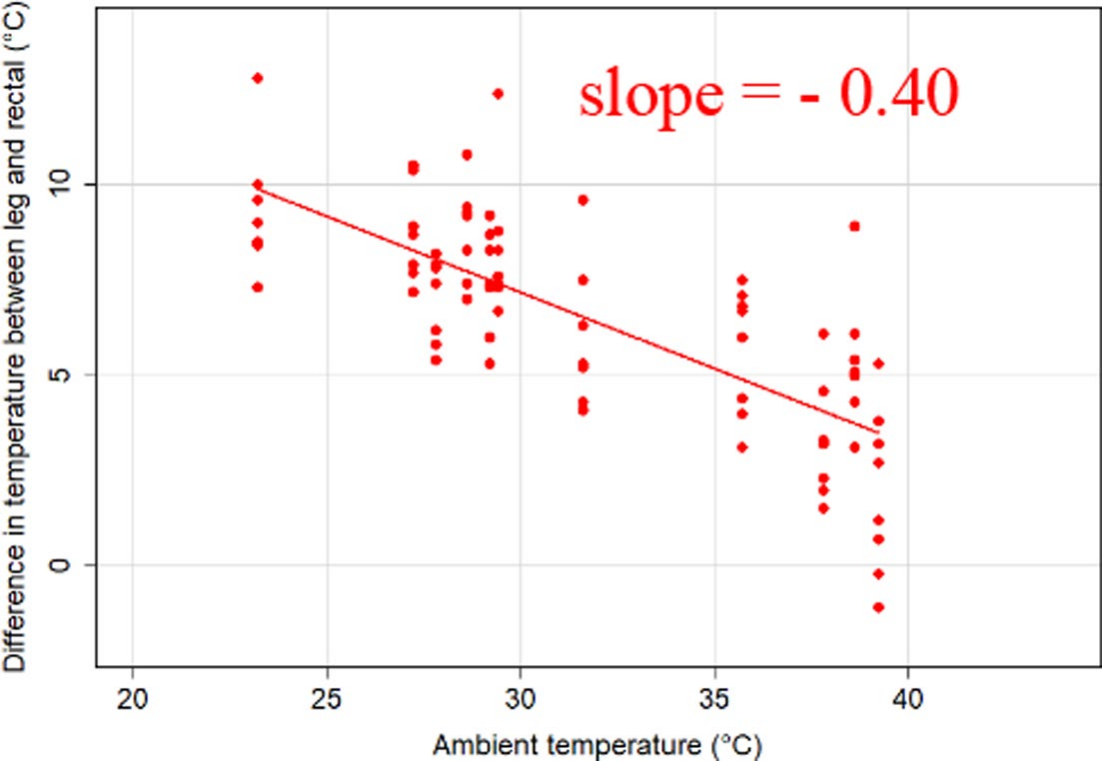	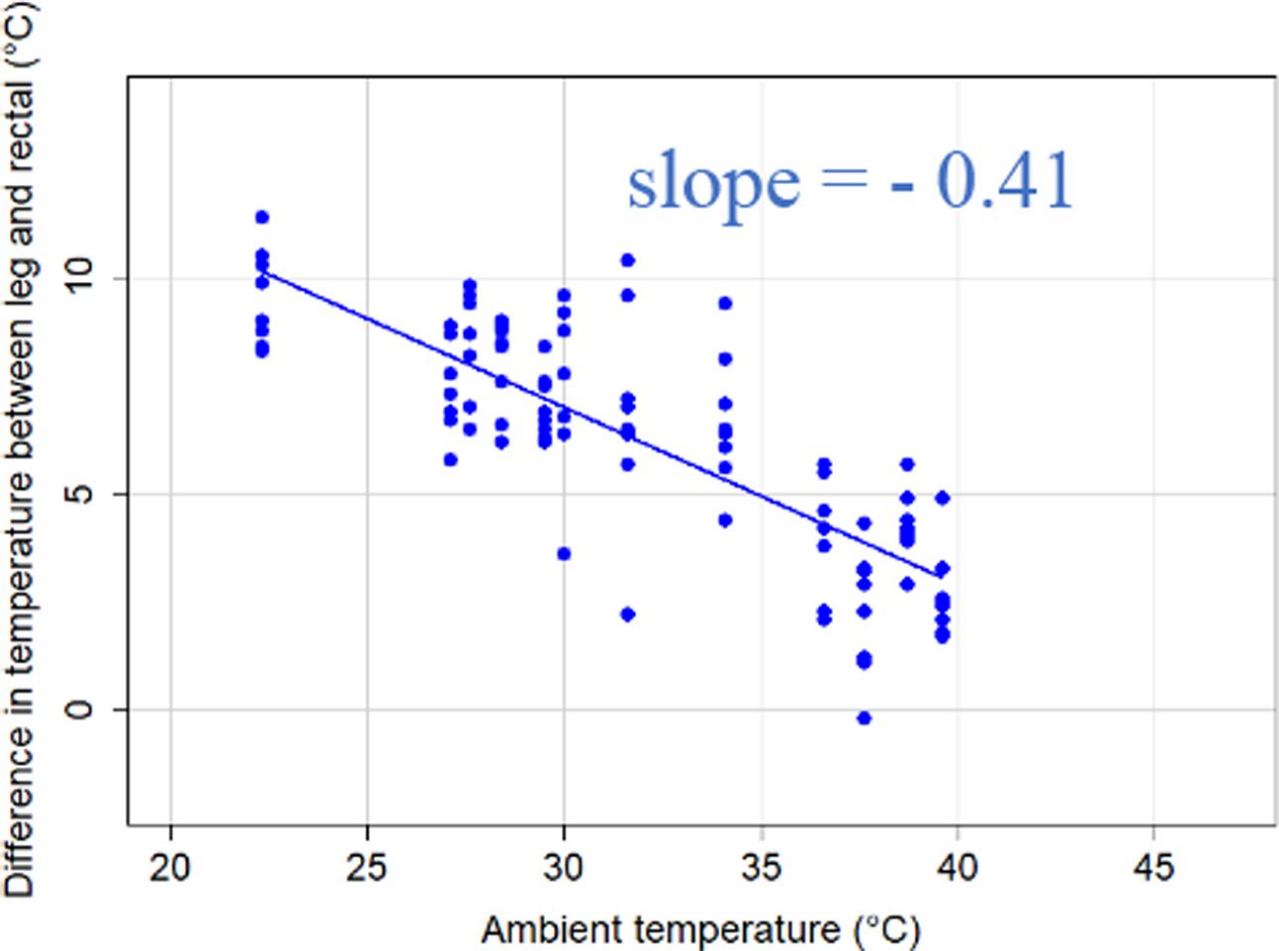

Models were fit without the extreme ambient temperature value of 46.4°C.

The association between the gradient between RT and ST during the daytime and the ambient temperature was significant (*p* < 0.001) in all measuring sites. The temperature gradient, and therefore the heat dissipation capacity through conductive heat transfer, is maximal in thermoneutral conditions and decreases with increasing ambient temperature. The average rate of decrease is reflected in the slope of the regression equations, which is an estimate of the average decrease in the temperature gradient between core and surface per 1°C increase in ambient temperature. We assumed that regions with a broader range of gradient with higher sensitivity to the changes of ambient temperature and higher explanatory power are the major scenes of heat transfer. Based on the coefficients estimated by the mixed-effects models the ear, leg and scapula temperatures were considered more informative of the actual heat dissipation capacity of the animal than muzzle or eye temperature gradients. Unlike the other regions, the muzzle is hairless and usually wet, which promotes evaporative heat loss supporting lower ST and higher temperature gradient between its surface and the core (27). Heat dissipation through the muzzle is high, even at higher temperatures, however, the surface area of the muzzle is very small compared to the flank or limbs.

### 3.5. Predictive value of surface temperatures in assessing the risk of hyperthermia

**Figure 3:**
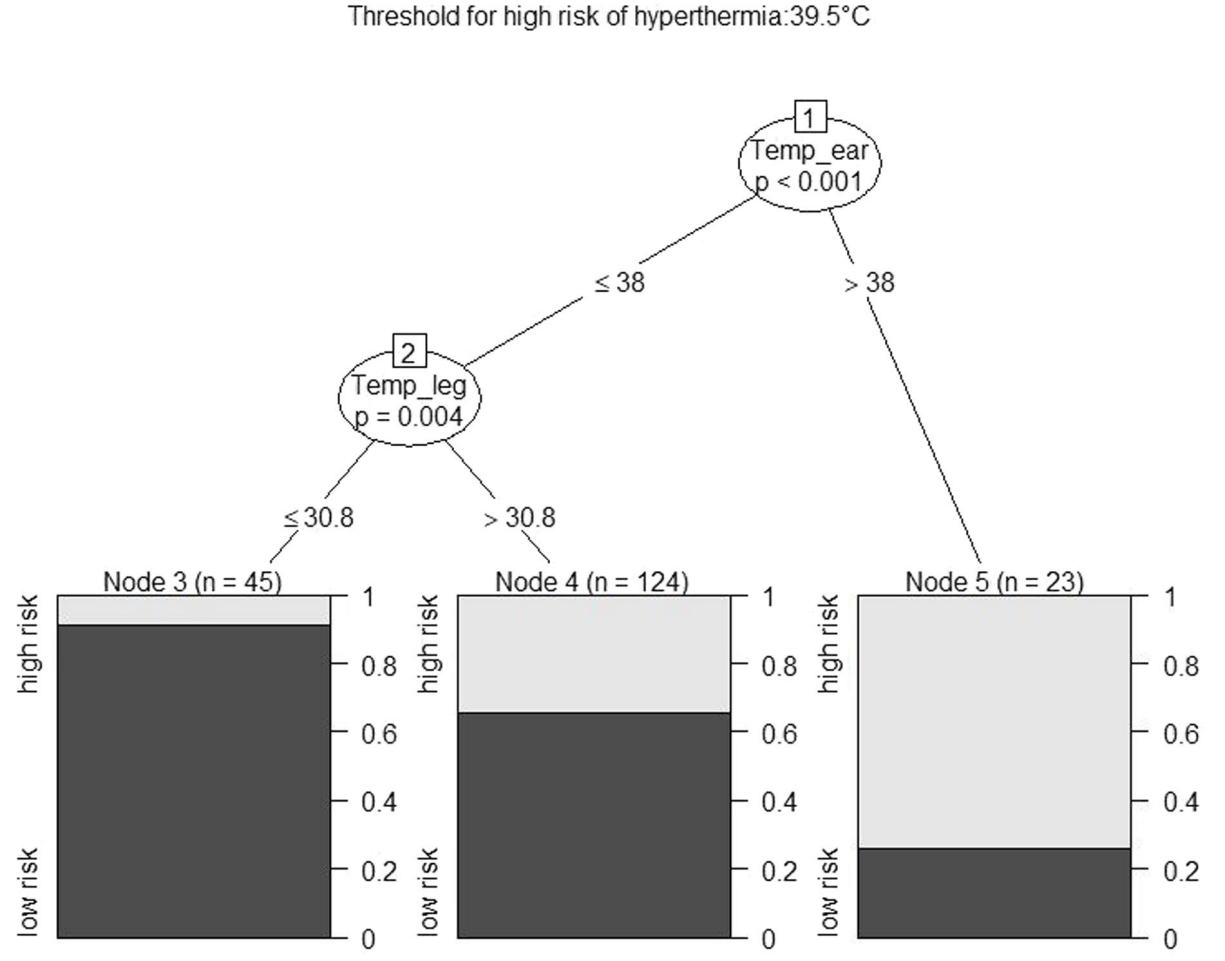
The output of a decision tree on classifying cases as being or not being at risk of hyperthermia (defined as a rectal temperature above 39.5°C). Nodes 1 and 2 indicate cutoffs, Nodes 3–5 indicate grouped observations and the probability of the outcome in the given node.

In the classification algorithm, the ear, the scapula and the leg temperatures were applied as explanatory variables to classify individuals as being at risk or having no risk of hyperthermia at a given time. Referring to the study of Piccione et al. (20) and the average rectal temperatures of the two groups, we have defined a high risk of hyperthermia as having a rectal temperature above 39.5°C. Figure 3 displays the cutoff points in skin temperatures that best separate between animals being or not being at risk of hyperthermia (*n* stands for the number of observations in the given group).

The figure shows that an ear temperature above or below 38°C and a leg temperature above or below 30.8 separate the cases by creating the greatest homogeneity in the outcome variable. The bar plots depict the frequency distribution of the outcome within that node. Predictions were made by classifying the observations to each of the „nodes” on the basis of the skin temperatures, and the outcome with the higher probability in the given node was assigned to that observation. The risk of hyperthermia that was predicted this way was compared to the risk that was assessed on the basis of the rectal temperature. The agreement with this approach was 72%. The figure shows that despite achieving fairly good homogeneity in animals with an ear temperature above 38°C or low risk of hyperthermia in animals with a leg temperature below 30.8°C, most observations (*n* = 124) were classified in a group that was heterogeneous in terms of the outcome.

With a similar approach, other thresholds of the risk of hyperthermia were also defined and tested for prediction accuracy. The results are summed up in Table 5.

**Table 5:** Thresholds for defining an animal being at risk of hyperthermia at a given time point and the accuracy of predicting the risk of hyperthermia on the basis of the skin temperature cutoff obtained from a decision tree algorithm.

Rectal temperature threshold (°C) for the definition of high/low risk of hyperthermia	Skin temperature cutoff (°C) in predicting high/low risk of hyperthermia	Predictive ability of the model
39.0	Leg skin temperature > / < 31.9.	71.3%
39.1	Leg skin temperature > / < 31.9.	69%
39.2	Leg skin temperature > / < 31.9.	67%
39.3	Ear skin temperature > / < 32.6 Leg skin temperature > / < 35	63%
39.4	Leg skin temperature > / < 32	59%
39.5	Ear skin temperature > / < 38.0 Leg skin temperature > / < 30.8	72%

Table 5 shows that predictive ability and skin temperature cutoffs are very sensitive to the thresholds defining hyperthermia. Accuracy of the device, measuring distance, hair depth or color can also greatly influence the predictive ability of this approach. Another explanation for the low interpretability is that surface and core temperatures show less agreement in clinically healthy animals (10). Despite the significant fluctuations in ST and the consequent changes in heat dissipation capacity, the duration of the exposure to high temperatures during the day did not induce large variability in the rectal temperature. According to Dado-Senn et al., “regardless of thermal exposure, the strongest correlations occurred between unshaved ST and ambient temperature or THI, and thus ST may be the most optimal indicator of heat stress for dairy calves in a shaded, subtropical climate” (15), or “monitoring thermal discomfort and potential heat stress *via* surface level infrared temperature is still effective and becoming increasingly common” (16). We argue with these statements. The authors also showed that “assessment of skin temperature requires caution, as it is not a good predictor of core body temperature (i.e., RT), which is the standard for assessing homeothermy and heat stress” (15). Our results also showed that the ST change quickly with the ambient temperature, and their values are small to predict the RT in heat stress. Further studies are required to establish the physiological thresholds of ST on different body parts in different thermal conditions to effectively use IR thermometry to detect heat stress in calves.

## 4. Conclusion

In conclusion, body surface temperatures are primarily related to ambient temperature in calves rather than rectal temperature. The predictive value of body surface temperatures for heat stress or rectal temperature is low for infrared thermometry. It follows that infrared thermometry is not suitable for practical use in the field. Calf body temperature can still be safely determined by rectal temperature measurement.

## Data availability statement

The raw data supporting the conclusions of this article will be made available by the authors, without undue reservation.

## Ethics statement

The animal study was reviewed and approved by Pest County Government Office, Department of Animal Health (Permit Number: PE/EA/1973–6/2016). Written informed consent was obtained from the owners for the participation of their animals in this study.

## Author contributions

MB, LK, and VJ proposed and designed the study. LFK, LK, and VJ performed measurements. MB performed the statistical analysis. MB and VJ drafted the manuscript, interpreted the results, and were assisted by LFK and LK. All authors contributed to the article and approved the submitted version.

## Funding

The authors are grateful for the support by the OTKA Research Scholarship of the National Research, Development and Innovation Office (Budapest, Hungary; grant no.: K-134204). LK was supported by the following grants: 2020–1.1.2-PIACI-KFI-2020-00109, 2020–1.1.2-PIACI-KFI-2021-00290, and GINOP PLUSZ-2.1.1–21-1366973 by the National Research, Development and Innovation Office (Budapest, Hungary).

## Conflict of interest

The authors declare that the research was conducted in the absence of any commercial or financial relationships that could be construed as a potential conflict of interest.

## Publisher’s note

All claims expressed in this article are solely those of the authors and do not necessarily represent those of their affiliated organizations, or those of the publisher, the editors and the reviewers. Any product that may be evaluated in this article, or claim that may be made by its manufacturer, is not guaranteed or endorsed by the publisher.
